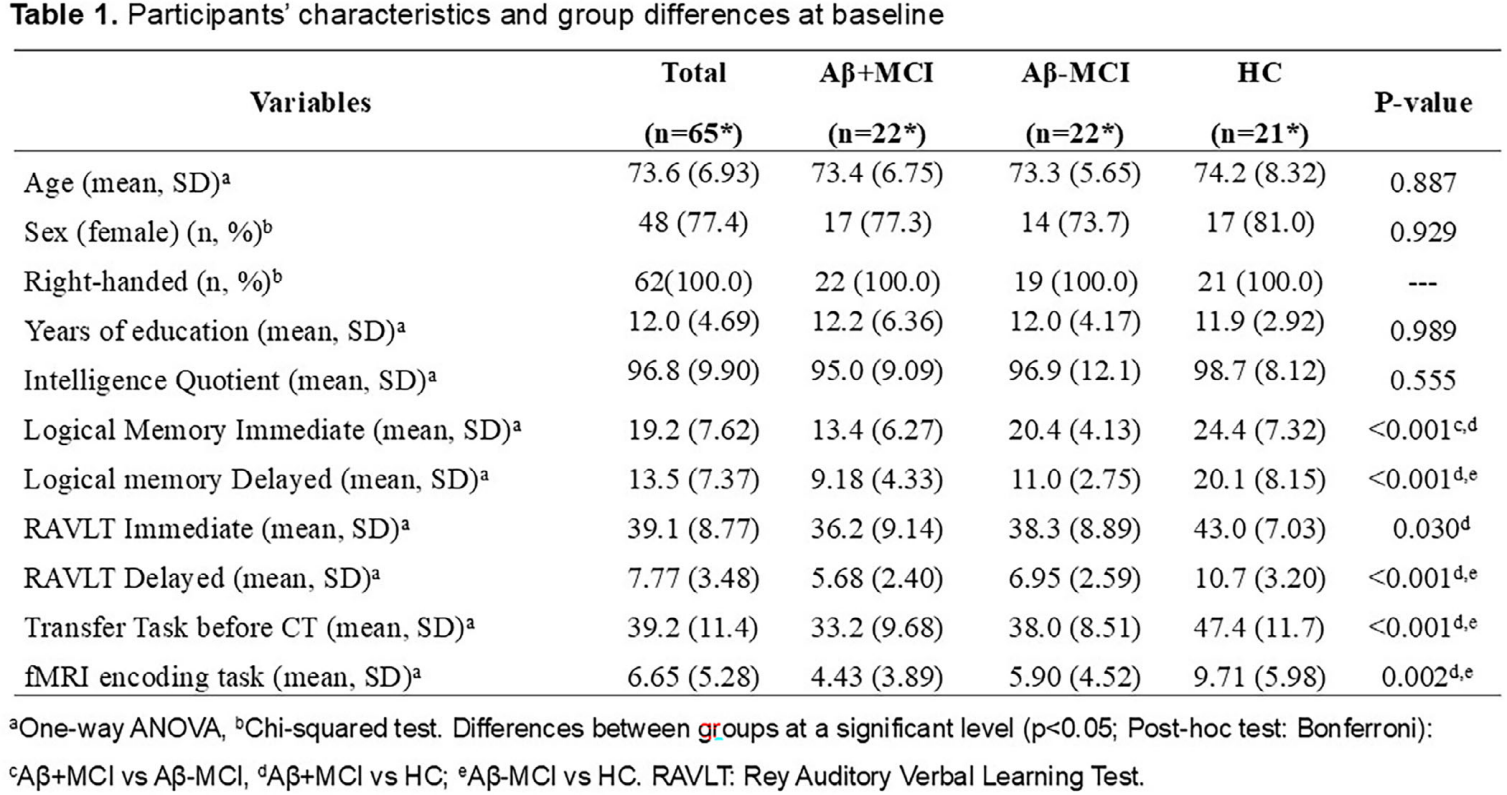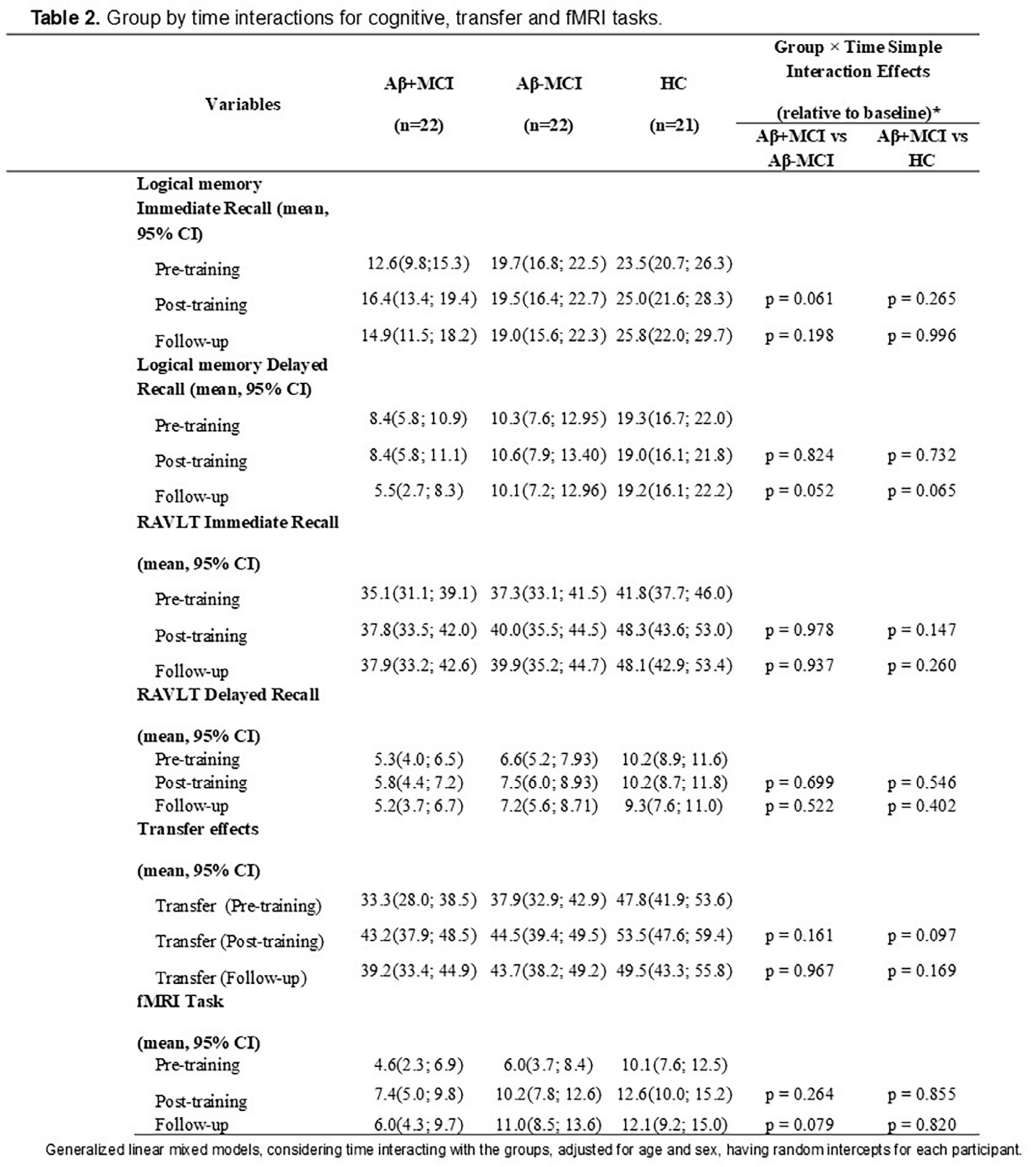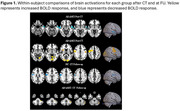# Effects of Cognitive Training on Cognition and Brain Activity in MCI with and without Amyloid Pathology and Healthy Controls

**DOI:** 10.1002/alz70856_105604

**Published:** 2026-01-09

**Authors:** Eliane C Miotto, Alana C Batista, Paulo R Bazan, Geise Aline A Silva, Raquel S Brandão, Daiane C Pinson, Maria da Graça, M. Martins, Luciana Cassimiro, Maria do Rosario C Rosa, Geraldo Busatto Filho, Arthur Coutinho, Eduardo Sturzeneker Tres, Ricardo Nitrini, Sonia Maria Dozzi Brucki

**Affiliations:** ^1^ University of São Paulo, São Paulo, São Paulo, Brazil; ^2^ University of Sao Paulo, sao paulo, SP, Brazil; ^3^ University of São Paulo, Sao Paulo, SP, Brazil; ^4^ University of São Paulo, São Paulo, SP, Brazil; ^5^ Universidade de São Paulo, São Paulo, São Paulo, Brazil; ^6^ Universidade de Sao Paulo, Sao Paulo, SP, Brazil

## Abstract

**Background:**

Cognitive training (CT) showed benefits in mild cognitive impairment (MCI). However, there is a lack of studies exploring its effects on positive (Aβ+) and negative (Aβ‐) amyloid MCI individuals. This study investigated the effects of CT on cognition and its brain correlates in those individuals and healthy controls (HC).

**Method:**

This prospective, blinded RCT (ClinicalTrials.govNCT03263247) included 65 participants: ^11^C‐PiB‐PET Aβ+MCI (*N* = 22) and Aβ‐MCI (*N* = 22), and HC (*N* = 21) using Petersen´s criteria. They completed Cognitive, Transfer tasks and fMRI scans before, after and 9‐12 month's follow‐up (FU) following 6 CT sessions using visual imagery to remember newspaper reports. We used general linear mixed models with time by group interactions, adjusted for age, gender and multiple comparisons. Neuroimaging data were acquired during a newspaper encoding fMRI task using 3T MRI and FSL for data analysis.

**Result:**

Before CT, both Aβ+ and Aβ‐MCI groups performed worse than HC on Logical Memory, RAVLT, Transfer and fMRI tasks. Notably, the Aβ+MCI showed lower performance compared to Aβ‐MCI on Logical Memory (Table 1). After CT, the latter difference disappeared as well as between HC and both Aβ+ and Aβ‐MCI groups on Transfer and fMRI tasks, although the magnitude of improvement was larger for HC and Aβ‐MCI. At FU, Aβ+MCI exhibited a mild decline in memory performance, while the other groups maintained their improvements (Table 2). Neuroimaging data after CT revealed increased activation in the right prefrontal, bilateral pre‐ and postcentral gyri and occipital cortex for Aβ+MCI. In contrast, Aβ‐MCI displayed bilateral temporal cortex reduced activation. At FU, Aβ+MCI showed increased right cerebellum activation, while HC exhibited decreased right pre‐ and postcentral gyrus activation (Figure 1).

**Conclusion:**

These findings suggest beneficial effects of CT across all groups in memory, fMRI and Transfer tasks. At FU, those gains persisted for the HC and Aβ‐MCI and slightly declined for the Aβ+MCI. Neuroimaging results indicated distinct patterns of increased and decreased activation across frontal, parietal, temporal and occipital regions among the groups. These outcomes suggest different compensatory cognitive and neural mechanisms of CT for Aβ+MCI in comparison to Aβ‐MCI and HC.